# Knowledge, Attitude and Practice of Community Pharmacists Regarding Antibiotic Use and Infectious Diseases: A Cross-Sectional Survey in Hungary (KAPPhA-HU)

**DOI:** 10.3390/antibiotics9020041

**Published:** 2020-01-21

**Authors:** Márió Gajdács, Edit Paulik, Andrea Szabó

**Affiliations:** 1 Department of Pharmacodynamics and Biopharmacy, Faculty of Pharmacy, University of Szeged, Eötvös utca 6, 6720 Szeged, Hungary; 2 Department of Public Health, Faculty of Medicine, University of Szeged, Dóm tér 10, 6720 Szeged, Hungary; paulik.edit@med.u-szeged.hu (E.P.); szabo.andrea@med.u-szeged.hu (A.S.)

**Keywords:** community pharmacists, antibiotics, non-prescription, dispensing, knowledge, attitudes, practice, responsibility

## Abstract

One of the key drivers for the emergence and spread of antimicrobial resistance (AMR) is non-prudent antibiotic (AB) use, which results in selection pressure towards relevant bacteria. Community pharmacists have pivotal roles in facilitating the prudent use of ABs that have been demonstrated by several studies worldwide. The aim of our present study was to evaluate the knowledge, attitude and practice of community pharmacists related to AB use and infectious diseases in Hungary. A descriptive cross-sectional survey was performed among community pharmacists in Hungary with the use of an anonymous, structured and pilot-tested questionnaire. Data collection ran between January 2016 and January 2018; n = 339 community pharmacists nationwide were approached with our questionnaire, out of which 192 filled out our survey. Hungarian pharmacists have appropriate knowledge regarding ABs and antimicrobial therapy, and they realize the public health impact of the growing AMR. Twenty-five percent of participants admitted to giving out non-prescription ABs at least once in the last year. The age and presence of board-certified specializations were shown to be significant factors of self-perceived knowledge and professional attitudes. Educational strategies and interventions specifically aimed at focusing on identified shortcomings and changing certain attitudes could substantially improve AB dispensing and AB use, in addition to minimizing resistance.

## 1. Introduction

Since the end of the 20th century, antibiotics have started to lose their efficacy. Due to the rapid emergence of antibiotic (AB)-resistant bacteria (especially in Gram-negatives [1,2]) and a dwindling AB pipeline (principally drugs that would be useful in primary care [3,4]), the therapeutic armamentarium of physicians has narrowed considerably [5,6,7]. Multidrug-resistant (MDR) bacteria are associated with prolonged hospital stays, decreased quality of life (QoL), additional costs for the healthcare infrastructure, and excess mortality [8,9,10]. In fact, in 2011, the World Health Organization (WHO) decided on antimicrobial resistance (AMR) (Combat Antimicrobial Resistance: No Action Today, No Cure Tomorrow’) as the theme for World Health Day (seventh of April), signifying the magnitude of this phenomenon and as being one of top three threats to humanity [11].

All healthcare professionals (HCPs), including nurses, technicians, pharmacists and physicians, have a responsibility in their clinical practice in keeping ABs effective [12,13,14,15,16,17,18]. Around 90% of AB consumption in human medicine occurs in outpatient settings; therefore, it is imperative to administer these drugs only in cases where it is appropriate [19,20,21]. Community pharmacists (CPs) are medical professionals in the primary healthcare system whom are often termed as the “first and last” healthcare providers [22]. They have significant roles in medicine provision, primary prevention, patient education, lifestyle-advice and safety monitoring (e.g., pharmaco-vigilance studies) [23,24,25]. In addition, due to the over-exerted primary care infrastructure of many countries (including Hungary), they may be the first point-of-contact with the healthcare system for many patients. CPs have pivotal roles in facilitating the prudent use of ABs [23,24,25]. They are well placed enough to advise patients about the correct application of ABs, the importance of intake regularity or when to finish therapy, interactions with food or other medications, possible adverse events, and, most importantly, they can highlight the importance and consequences of AB misuse and the basics of antimicrobial resistance [23,24,25,26].

Qualitative and quantitative levels of AB-consumption vary greatly in different countries in Europe; however, a north-to-south and west-to-east gradient of higher consumption rates has been observed [27]. The quantitative AB-consumption rate of Hungary is on the lower end of the spectrum, though the proportion of broad-spectrum agents used is the highest among EU Member States [27,28]. Around 93% of ABs are obtained from community pharmacies in Hungary (based on the results of the recent Eurobarometer report) [28]; however, a recent study demonstrated that ~2% of ABs in the country are from non-prescription sources and that non-prescription AB-sales have tripled over the last decade [29]. Additionally, a recent study in Hungary showed that a lack of primary care availability (which is a developing problem) leads to the over-prescription of ABs [30]. To the best of our knowledge, there has not been a single study to comprehensively assess the attitudes of community pharmacists related to their roles in prudent AB use in Hungary. The aim of our study was to evaluate the **K**nowledge, **A**ttitude and **P**ractice of community **Ph**armacists related to **A**ntibiotic use and infectious diseases in **Hu**ngary (or **KAPPhA-HU** Study).

## 2. Results

### 2.1. Demographic Characteristics

**Figure 1:**
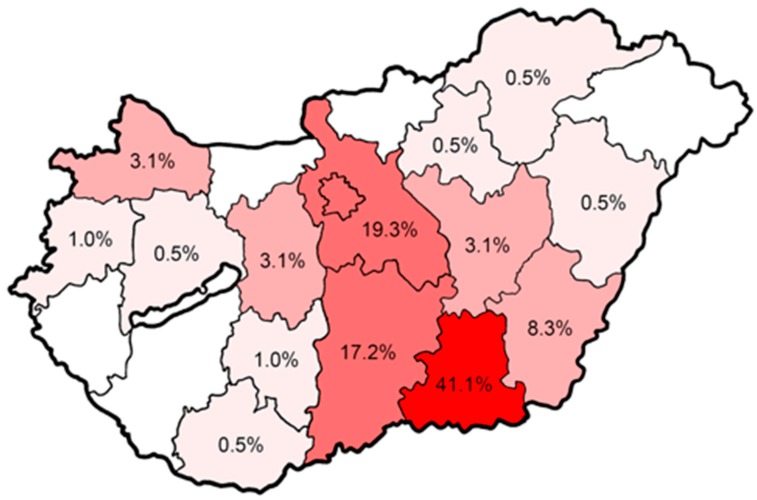
Geographical distribution of participants (n = 192) in the different counties in Hungary.

At the time of the study, n = 5575 CPs (4566 full-time and 1027 part-time or as substitute pharmacists) were employed in Hungary. A total of 339 CPs nationwide were approached with our questionnaire during post-gradual training sessions at three different institutions throughout Hungary, out of which a total of 195 CPs chose to participate in our survey (corresponding to a response rate of 57.5%). Three respondents (1.5%) were excluded due to incompletely filled out questionnaires; therefore, n = 192 were included in the final analysis. The majority of the respondents were from the Csongrád (41.1%), Pest (19.3%) and Bács-Kiskun (17.2%) counties, and there were no participants in five out the nineteen counties in Hungary (Figure 1). Out of the 192 participants, 69.8% (n = 134) were female and 41.1% were over 35 years of age (Table 1). The median age of the respondents was 30.5 years (average: 36.0 ± 11.7; range: 24–76 years), with younger participants in higher proportion (24–35 years: 58.9%). Almost two-thirds of pharmacists did not have a specialization, while 34.4% of respondents were board-certified pharmaceutical specialists (BCSPs); most of the specialized degrees concerned pharmacy operation and management (60 out of 66) (Table 1).

### 2.2. Self-Perceived Knowledge-Level of CPs Regarding ABs and Infectious Diseases

In the questions related to self-perceived knowledge (QK1–QK3 [questions related to knowledge], presented in Table 2.), the respondents received one point for each ‘true’ answer, totaling between 0 and 3 points. The average number of points during the analysis was 2.27 ± 0.86 (3.6%, 16.7%, 28.6% and 51.1% of respondents received 0, 1, 2 and 3 points, respectively). While most of the CPs (>90%) considered their knowledge on AB therapy appropriate, this ratio decreased considerably when the same question was asked regarding the knowledge related to medical microbiology and infectious disease-epidemiology (70.8%) or bacterial resistance mechanisms (67.2%).

### 2.3. AB Utilization

The participants were asked to assess the AB utilization (QU1 [questions about AB utilization]) and the ratio of AB prescriptions on a pre-determined percentage scale as compared to the total prescription drug traffic in their respective pharmacies at which they are currently employed; the results are presented in Table 3. Individual pharmacies may significantly differ in the number, however, and, based on the estimations of the majority of respondents, around 5%–20% of prescriptions are for ABs (according to 65.3% of respondents).

### 2.4. Theoretical Attitude

The theoretical attitudes of CPs were assessed by using eight questions (QTA1–QTA8) (Table 4). All respondents (100%) agreed that the inappropriate use of ABs is an issue in healthcare (QTA1). In another question, the opinions of the respondents were asked on the practice of other colleagues (the statement was “I believe that it is problematic that there are pharmacists who dispense antibiotics when the patients request them without a prescription,” QTA2); 87.0% (n = 167) respondents chose “True,” while 5.2% (n = 10) and 7.8% (n = 15) chose “False” and “I don’t know/Unsure” of the answer, respectively. From QTA3, the distribution of the responses to individual questions are presented in Table 4. Opinions about AB funding policy were divisive, as 27% of the pharmacist disagreed, 47.4% agreed, and 28.6% were unsure (QTA3); 93.7% of the respondents agreed that ABs are medicines of special importance (QTA4); 77% of the respondents were aware that inappropriate AB therapy causes significant surplus health costs, but 18.8% did not know this (QTA6); 92.7% agreed that university education should be more prominent about ABs (QTA7); and 79.1% of the pharmacists had the proper knowledge about the fact that the use of ABs in animal husbandry as growth promoters is just as important in the development of bacterial resistance, as their inappropriate prescription/consumption in health care, but 20.8% had no idea or disagreed with the fact (QTA8).

### 2.5. Practical Attitude

When asking about non-prescription AB-sales, respondents were classified as “low-risk” or “high-risk” pharmacists during analyses; 74.0% of CPs stated that they had never given out ABs without a prescription of a physician (QPA1) (Table 4) (“low-risk pharmacists,” excluding dispensing to other healthcare-professionals in exchange to presenting relevant professional licenses). In contrast, 21.9% of participants admitted to giving out non-prescription ABs at least once, while 4.1% had given out ABs at least five times in the last 12 months (“high-risk pharmacists”). Because pharmacists often get feedback from patients, they can evaluate their successfulness after giving advice, and 79.2% of them reported that patients happily accepted the counsels (QPA2). Positive reaction can further motivate a pharmacist in their everyday work. Quite a big proportion, 85.4% of the respondents felt obligated to inform those patients who requested ABs without perception of their risk, though 12% believed the opposite (QPA3). The majority of pharmacists (85.9%) perceived that patients enter a pharmacy with a lack information and what should have been given by physicians previously; therefore, pharmacists need to set more time for patient education (QPA4). Pure practical competency was measured by the percentage of pharmacists who recommend probiotics and who detail the proper use of ABs for patients purchasing prescribed ABs, which had very high response rates, with 92.2% and 98%, respectively (QPA6 and QPA7).

### 2.6. Attitude Towards Prevention

The majority of pharmacists (88.6%) realized that the media does not devote enough energy to disseminate information on infectious diseases (QPr1) (Table 4). It seems from the answers given to the questions studying the attitudes of respondents regarding their patient educator roles that there is an agreement and a readiness among CPs, as more than a 90% agreement was measured for QPrA2–QPrA4 question. Generally, they believed in the power of patient education, that they can influence the patients’ approaches to infectious diseases, and that they need to provide lifestyle advice as well. Additionally, a big proportion of pharmacists (79.1%) felt that the proper use of ABs would be greater if pharmacists had time to perform their pharmacological care duties (QPrA5).

### 2.7. Professional Attitude and Role Expansion

The pharmacists were asked about potential expansion of their roles in the prevention of infectious diseases, the therapy of infectious diseases and their pharmaceutical profession overall (QPh1–QPh3) (Table 4); 46.4% of respondents were against the idea or was unsure about therapeutic decisions on uncomplicated infections (e.g., urinary tract infections and bacterial tonsillitis), while 60.5% were against pharmacists in the management and administration of vaccinations (respondents without a specialty and >35 years of age were more likely to have a negative attitude; *p* = 0.011 and *p* = 0.013, respectively). However, 95.3% of the respondents stated that medicine-related counseling by CPs is just as important as the physician’s recommendations. A significant correlation was found between the self-perceived knowledge of CPs and their age (lower number of points if ≥35 years of age; *p* = 0.026) and specialization (board-certified pharmaceutical specialists (BCPSs) had a higher number of points; *p* = 0.048). Respondents with no specialization (*p* = 0.011) and over 35 years of age (*p* = 0.013) were more frequently high-risk pharmacists among the surveyed population. Of note, responses to QPA5 were especially daunting, as 43.8% admitted that the temperament of patients influences their everyday dispensing practice; this phenomenon was more prominent in pharmacists without specialty training (*p* = 0.002) and respondents ≤35 years if age (*p* = 0.009).

### 2.8. Correlational Analysis between Self-Perceived Knowledge and Certain Attitudes

The results of a correlational analysis of responses pertaining to theoretical–practical–preventive attitudes are shown in Supplementary Tables S2–S4. Self-perceived knowledge showed very few significant and weak correlations with theoretical, practical or preventive attitudes (Supplementary Table S2). Proper knowledge about the pathomechanisms and preventions of infectious diseases went together with the higher motivation to get to know new ABs and the proper practice of the less likely dispensing of ABs without a prescription; however, this knowledge was negatively correlated with a recognition of the poor public information of the media about infectious diseases, and it was less likely to be believed that pharmacists can form a patients’ approach about infectious diseases. Proper knowledge regarding bacterial resistance came along with the appropriate theoretical and practical attitudes of not dispensing ABs without a prescription and more likely providing lifestyle advice in addition to other recommendations.

All measured items of the theoretical attitude were positively and significantly associated to several items of the practical attitude (Supplementary Table S3), with an especially strong association to better acquaintance with new ABs, which resulted in the CPs less often dispensing ABs without a prescription; with more acceptable pharmaceutical advice; with patient education when requiring ABs without prescription; or with more detailed advice about the proper use of ABs. It could be also seen from the correlational coefficients that any improvement in theoretical knowledge could eventually lead to the more frequent—and hopefully efficient—informing and educating a patient who wants to access to AB without medical prescription. However, better theoretical knowledge did not influence the temptations caused by the patients’ behavior/personality and did not improve the recommendation frequency of probiotics.

A better theoretical attitude positively and significantly correlated with good preventive attitudes, as well (Supplementary Table S3). The attitudes were particularly strong in the areas of the awareness of the fact that if they themselves or other pharmacists dispensed an AB without prescription is a problem, knowing the fact that animal husbandry has also a role in AB resistance called for the preventive attitude of understanding the efficiency of patient education and pharmacists in the prevention of infectious diseases, and offering healthy lifestyle recommendations as a must. Only appreciating the role of media in the prevention of infectious diseases was independent form the level of theoretical attitude.

An improved preventive attitude was related to a better practical attitude in general (Supplementary Table S4). In the preventive domain, the confident belief that pharmacists could really form the patients’ attitude toward infectious diseases positively affected those practical behaviors, as resisting the patients’ temperament when making decisions in dispensing, when giving acceptable advice, or when educating the patients who ask for AB without prescription. This latter, was positively influenced by other preventive attitudes, as well, like understanding the positive role of patient education or with the highest correlational coefficient and with the strongest relation by accepting the additional task of a pharmacist to provide recommendation on a healthy lifestyle. Interestingly, some of the appropriate practical attitudes went together with the over-evaluation of the role of media (negative correlation) in raising public awareness about infectious diseases.

## 3. Discussion

The present nationwide survey aimed to explore CPs’ knowledge, attitude and practice regarding ABs and infectious diseases, as well as their relationship with the dimension of professional responsibility related to the spread of AB resistance in Hungary. Our results showed that respondents ≥35 years of age and pharmacists without specialization were less confident about their roles and knowledge regarding ABs and infectious diseases. The majority of respondents had a high level of theoretical knowledge regarding antimicrobials and resistance. The vast majority (87%) of our interviewed pharmacists agreed that other pharmacists should not dispense ABs without a medical prescription, while almost the same amount of them (84.9%) evaluated themselves, as well, as causing public health problem if they do this malpractice. Despite this, 26% of the interviewed pharmacists confessed that they had already dispensed non-prescribed ABs. We found that almost half of the pharmacists perceived that the patients’ personality and behaviors could influence their dispensing habits. According to the correlational analysis, the manipulating nature of the patients’ temperament on dispensing pattern could not be influenced by any theoretical or preventive item, although pharmacists’ knowledge and attitude were strongly associated with dispensing a non-prescribed AB. Around half of the respondents showed resistance towards the potential expansion of the professional role of CPs.

The roles of healthcare professionals in the fight against AB resistance were emphasized in multiple reports by the WHO. The requirements on the knowledge-level of healthcare professionals were re-iterated in the ‘Competency framework for health worker’s education and training on antimicrobial resistance,’ which details specific points of competence for all healthcare professionals working in different settings [31]. The most recent report was published by the European Centre for Disease Prevention and Control [ECDC], which (similarly to the present study) aimed to assess knowledge, attitudes and behaviors on ABs, AB use and AMR in the European Union/European Economic Area [EU/EEA] [32]; out of the 18,365 HCPs in different healthcare settings, 17.7% were pharmacists. In the report, most (89%) respondents agreed that their practice of the prescribing/dispensing/administering of ABs directly affected the emergence and spread of AB-resistant bacteria; in addition, knowledge on ABs was measured with seven questions, and Hungarian HCPs were among the EU countries where less than 50% of respondents answered all questions correctly [32].

### 3.1. Knowledge-Level of CPs

Considering the theoretical knowledge, every respondent in our study unanimously agreed that inappropriate AB use is a major health problem, and almost everyone considered ABs as medicines of special importance. In a Portuguese survey, 100% of pharmacists declared that AB resistance is an important public health issue [33]. However, Australian hospital pharmacists reported ambivalent views towards the significance of AB resistance and regarded optimizing AB use as a low priority [34]. In our study, 18.8% of the respondents thought that AB misuse could not cause surplus health cost, this group comprised 30.4% of the Albanian pharmacists who were interviewed in a previous study [35]. According to our results, the majority of pharmacists knew that AB misuse in animal care can also contribute to AB resistance. The external responsibility of veterinarians has also been perceived by other studies [16,33].

### 3.2. Non-Prescription AB Use

Non-prescription (OTC) AB use and antibiotic self-medication (SMA) is one of the main factors in the irrational use of ABs and the development of MDR bacteria, so controlling AB use may present as a viable means of intervention [36,37,38]. Our results (e.g., 26% of the interviewed had dispensed non-prescribed ABs) are comparable with those found by Vazquez-Lago et al., as they found that 5%–20% of the pharmacists dispensed non-prescribed ABs [39]. A very similar proportion of the Albanian pharmacists, 89%, thought that ABs should not be given out without a prescription [35]. Other European studies have found that pharmacist occasionally dispense non-prescribed ABs [40,41]; however, there are big differences within Europe, e.g., in Spain, though the unlawful dispensing of ABs without a medical prescription is a common practice, as 65% of the responding pharmacist reported this bad practical attitude. This malpractice has not been correlated with age, gender, or years of professional experience [42]. In a representative Portuguese study, 49% of the pharmacy workers had a propensity to dispense ABs without medical prescription if the patient was known to the pharmacist [33]. The finding of another Portuguese study revealed the same to what we found, namely that elderly generations were more likely have dispensed an unprescribed AB [43]. This may be because elderly pharmacists realize the impact of sales levels on their salaries, and they feel a priority to sell the AB.

The main reasons for the malpractice of non-prescription AB-dispensing were summarized by Vazquez-Lago et al. [39]: the study group had defined reasons such as economic/patient-specific (a.: pressure from patients to have their complaints rapidly resolved; b.: fear of losing loyal customers, especially in smaller towns; c.: fear of rejection by common people with not meet expectations; and d.: pressures from pharmacy owners), internal/pharmacist-specific (bad habits during dispensing and a lack of continuous professional development) and external (indifference of physicians regarding patient follow-up, a lack of communication, no enforcement on the part of the authorities). These findings have been reinforced by several other publications [33,34,35,36,37,38,40,41,42]. Recently, pharmacists have tended to shift the responsibility of the problem of antimicrobial resistance to external sources, such as patients, physicians, other pharmacies and veterinarians [33,43,44].

Patient demand was reported by CPs several times as being one of the greatest barriers to improve AB utilization [45]. In developed countries, like Hungary, the main reason for OTC ABs is patient demand and a fear of losing customers of the business. This is proven by our results, as one of the most remarkable findings was that almost half of the pharmacists perceived that the patients’ temperament could influence their dispensing habits, especially those with lack of experience and those who were less qualified. A Portuguese survey identified complacency as the primary factor (patient has difficulty/shortage of time or money in obtaining medical care or they can acquire in another pharmacy [33]). The pressure of losing patients because of competition between pharmacies could be minimized by changes in the healthcare-system, just like in the UK or Australia, where patients after visiting their allocated general practitioners, can get medicines dispensed from an adjacent pharmacy [46]. Maybe, if pharmacists were more aware that dispensing ABs is part of their social responsibility, just like in the UK [47], the status of Sweden could be reached, where the public trusts HCPs more if they do not prescribe ABs. Thus, it should be highlighted that CPs (especially in countries where their roles do not include prescribing/de-prescribing) alone are unable to promote appropriate AB use if GPs still supply patients with AB-prescriptions in inappropriate situations [33,40,41,42,43,46]. Of local relevance, a study by Gajdács et al. regarding the attitudes of GPs in the Csongrád county of Hungary found that around 80% of GPs were satisfied with their knowledge regarding infectious ailments and AB therapy, while only 48% were confident about their knowledge on AB-resistance; in addition, 25% of respondents stated that the personality and behavior of patients significantly influenced their prescribing practices [48]. However, along with our results, either any higher theoretical knowledge or any improved preventive view ended in one of the most important proactive practical behaviors of pharmacists, as they more likely educated those patients who were expected to have access ABs without a medical prescription. We found that pharmacists’ knowledge and attitude were associated with the dispensing of a non-prescribed AB. Zapata-Cachafeiro concluded the same; additionally, indifference, complacency, external responsibility and insufficient knowledge have all been identified as attitudes that increase the risk of this malpractice [42]. There have been promising results for reducing the prevalence of faulty dispensing, as it has been self-reported by pharmacists that patients who insist on obtaining an AB have been able to understand and stop insisting, though only after they explained the consequences of unnecessary use [43].

### 3.3. Role of CPs in Patient Education

Many strategies have been proposed to facilitate the prudent use of ABs in inpatient settings [49]. In contrast, stewardship practices are much less prevalent in outpatient medicine. Most strategies to improve prudent AB use emphasize the role of patient education (both by the use of banners and social media and by HCPs during visits to the physicians/pharmacies) [49]. According to international studies, doctors, pharmacists and nurses have been most frequently indicated as the most reliable sources of AB information [32]. Confirming this, the Special Eurobarometer on ABs (EBM 478) reported that in the last 12 months, 32% of respondents have taken ABs (33% in Hungary), and 22% of respondents had received advice from pharmacists on not taking ABs unnecessarily [28]. In the present study, almost 80% of respondents stated that a lack of time was to blame for inadequate patient education and prevention practices; the reason for this may be due to the over-exertion of these HCPs, as, based on projections of Hungarian National Healthcare Services Center, there would need to be 7500–8000 CPs employed for the unobjectionable provision of pharmaceuticals in primary care [50]. The present study revealed that the patient-educator role was very much emphasized by the Hungarian pharmacists, as, generally, they strongly trusted the power of patient education. Roque et al. also reported that all patients were advised by a pharmacist upon AB dispersion [33]. Everyday patient–pharmacist communication and pharmacist-involved public awareness campaigns have been proven to be efficient in several countries, e.g., antimicrobial consumption was successfully reduced with 60% by improved patient communication in a German campaign [51].

Professional pharmaceutical advice can really affect patients’ perceptions and attitude towards their illness and their perceived need for ABs. In a study done in London, it was revealed that those who received counselling from a CP displayed a better knowledge towards prudent AB usage than those who were exposed to an AB campaign [52]. This emphasizes the importance of pharmacists’ counselling on every AB prescription. However, for this, pharmacists must have the knowledge, attitude, self-confidence and communication skills to achieve this. If they believe that they can affect patients’ approach, they can more successfully acquire a good practical attitude. However, until pharmacists are perceived by the general population as “educated sellers” and their competencies are no longer underestimated by physicians, their confident standing by antimicrobial stewardship may be undermined [49,53]. A systematic review concluded that if pharmacists are involved in health promotion, then their reputation can improve [54].

There are several countries where physicians’ counselling practice is really poor or where, even if it appropriate, there are some topics related to infection prevention and control that are less likely to be counselled (food handling, household hygiene, proper coughing and hand-washing practices, and using hand sanitizer and antibacterial supplies) as physicians consider them less important in the prevention of antimicrobial resistance or they might expect that other health care professionals will do this. By our results, a very big proportion of respondents blamed physicians for insufficient patient education, and they sensed that they needed to expend surplus time to explain everything. Pharmacists’ negative views towards physicians are reinforced when they catch antimicrobial prescription errors, as, according to some reports, 19%–36% of prescriptions are illegible or incomplete [55]. This emphasizes the additional role of a pharmacist: a sentinel. There is evidence that clearly shows the importance of pharmacists in supporting GPs to foster appropriate the prescribing practice of ABs. This means structured visits by clinical pharmacists that target GPs when the pharmacist is a therapeutic adviser, an academic detailer, a reviewer of medication prescription and feedback provider [48]. All in all, general physician–community pharmacist close collaborations are essential and can be effective, as was confirmed by Vervloet et al. [56]. In the UK, the position of an antimicrobial pharmacist was introduced to support GPs’ decisions in AB prescribing [48].

### 3.4. Potential Expansion of Professional Role of Pharmacists

CPs were also surveyed regarding their potentially expanding professional role on administering vaccination and deciding on the pharmacotherapy of uncomplicated infections. Pharmacy-based influenza immunization services have been already running in England, Portugal, Belgium, Australia and some states of USA [24,25]. This could be a next step in involvement of pharmacists in direct patient care, and vaccination coverage has been proven to have been improved in these regions. There are some countries (Canada, New Zealand, UK) where pharmacists are legally allowed to prescribe antimicrobials in certain cutaneous or urinary tract infections, chlamydia or Lyme disease [57]. In USA, in the framework of collaborative practice agreement, pharmacists perform specific patient care functions, such as screening patients and prescribing antivirals in influenza season, immunizations, medication therapy management, and, when paired to a physician, implementing point-of-care tests and initiating appropriate therapy. Finally, this program has led to a more judicious use of antimicrobials in these areas [58]. In our present study, a significant portion of respondents were against the idea (>40% pro and >60% against), mainly among older HCPs. This may have been due to a variety of reasons: their inadequate training and competence level, their objections towards administering vaccines (technical aspects), their refusal of taking on additional responsibilities, and so on. However, the most probable reason was an external one, namely that the medical community (i.e., physicians) would not agree to such a change in the healthcare system in Hungary. This sentiment was verified by our previously mentioned study: while 72.3% of respondents believed that CPs have important roles in facilitating appropriate drug use, 77.1% of GPs objected to pharmacists making therapeutic decisions and administering vaccines [48]. Otherwise, the responding pharmacists stood by their competency and their importance in health care, as they evaluated their own counselling being equal to physicians’ instructions.

### 3.5. Influence of Personal and Professional Characteristics

While a correlation with age of the pharmacists seems logical (less time has elapsed since finishing training, and a decline in knowledge-level has been identified by many studies) [19,20,21], the correlation of roles and knowledge regarding ABs and infectious diseases with specialty training is less clear; most respondents had the “Pharmacy operation and management” specialty, which has been used as a prerequisite to become a Lead pharmacist in Hungary since 2015. The curriculum of the abovementioned training mainly involves managerial and economical competences, whilst topics of pharmacology and public health are not included; therefore it can be assumed that the acquisition of the specialty per se is not the reason for the better performance of the respondents; instead, their higher professional standing and confidence in their workplace may explain their better performance. Another possible explanation is that the importance of AB-resistance has taken center stage in the last 10–15 years due to increased media attention; however, pharmacists finishing their Doctor of Pharmacy (Pharm.D.) training over 15–20 years ago may have heard limited amounts of information on the topic. Adequate knowledge itself does not guarantee proper practices. This situation is called as the “theory–practice gap,” and many reports have suggested that practice is mainly influenced by the attitudes of HCPs [59].

In our study, almost everyone stated that they had the proper level knowledge about AB therapy, but they were less likely to be familiar with the pathomechanisms of infectious diseases and bacterial resistance. The same findings have been reported by other surveys, where majority of the pharmacists have been found to have an understanding of the problem of AB resistance and its effect(s) on public health, but they were not found to have a full understanding of how AMR develops and spreads [33,40,41,42,43,46]. According to our results, self-perceived knowledge on ABs and infectious diseases did not seem to be a good indicator of appropriate theoretical, practical or preventive attitude. Those who can realize their gaps in knowledge may have a good attitude towards proper AB use. An improved theoretical attitude was correlated to better practical and preventive attitude, and, vice versa, better preventive attitude was related to improved practical attitude. Even so, within the theoretical domain, getting acquainted with current and new ABs was the strongest influencer of a better practical attitude, which indirectly indicates that proper knowledge is indispensable for good attitude. Altogether, appropriately trained pharmacists are needed, and gaining a high level professional knowledge related to AB resistance at medical universities and strengthening the medical and pharmaceutical curricula have already been identified by several reports as important considerations for the future [24,32]. This was confirmed by our study, as almost all respondents agreed that university education should focus more on ABs.

Our study concluded that there is a complex relationship between the knowledge, attitude and practice of pharmacists on AB dispensing and willingness for counselling. The theoretical framework of connections between the three items have been used several times and discussed in publications in regard to AB utilization among physicians and pharmacists [33,40,41,42,43,46]. Our results reinforce the results published by Liu et al., as they also found that the knowledge, attitude, and practice of physicians toward AB prescription are closely linked to each other; however, improving knowledge itself is not enough because it is not always associated with intentions to change behavior [60,61].

### 3.6. Limitations of the Study

There are several limitations of this study that need to be addressed for transparency: (a.) There was a relatively low number of participants from the pool of practicing CPs (n = 192 vs. n = 5575); (b.) there were some counties in Hungary from which there were no participants in this study, so the findings may not represent the knowledge, attitude and practices of CPs in other parts of the country; (c.) the study sample was diverse in terms of participant’s age, years of experience, but >50% of pharmacists were 35 years old or younger, which may have resulted in a bias towards the opinions of younger healthcare-professionals; (d.) the questionnaire was self-administered, so data collection was prone to social desirability bias (tendency of respondents to choose answers that are socially acceptable instead of responses that mirror their true feelings or practices); (e.) during the survey, the knowledge of the relevant topics (AB therapy, infectious diseases, and resistance) was self-evaluated, but the correlation between the real knowledge and self-perceived knowledge was not tested; and (f.) the current survey could not measure the correlation between the practices of CPs with their attitudes—for the evaluation of this, simulated patient studies are needed [17]. Despite the mentioned limitations, some key concepts regarding CPs in Hungary have been identified. Furthermore, since the created novel survey instrument may facilitate the development of further studies.

## 4. Materials and Methods

### 4.1. Study Design, Questionnaire

A descriptive cross-sectional survey was performed among CPs in Hungary by using an anonymous, structured and pilot-tested questionnaire. The methods that were used during sample size calculation are described in the Supplementary Section S1.

An extensive literature review of similar surveys was conducted in the PubMed/MEDLINE database in order to identify potential questions for the development of the questionnaire that was used in this study. The content validation and internal consistency-assessment of the questionnaire are described in Supplementary Section S2. The final structured questionnaire consisted of 32 questions that covered three major areas: Section 1: demographic characteristics (four items: age, sex, qualification level, and location of the pharmacy); Section 2: questions about self-perceived knowledge and practice related to the dispensing of ABs (seven items, including subjective beliefs related to knowledge levels, ratio of AB-prescriptions in the pharmacy of interest, and the non-prescription dispensing of ABs) where the respondents had the option of choosing either percentages or were given the options of ‘Yes,’ ‘No,’ and ‘I don’t know/Unsure about the answer’ to choose from; Section 3: questions regarding attitudes and personal responsibility in the curbing of AB resistance (twenty-one items, including theoretical, practical and preventive attitudes, as well as views towards the current and emerging roles of CPs in medical interventions and pharmaceutical care), where a five-point Likert-scale ranging from “strongly agree” to “strongly disagree” was used to measure the responses of the participants. However, different items, belonging to the same attitude, were mixed in the questionnaire, and, upon analysis, they were examined together.

### 4.2. Data Collection and Statistical Analysis

Data collection for the survey was running between January 2016 and January 2018. Participants were informed about the purpose, benefits, and risks of the study, and each participant provided written informed consent. Participants completed the questionnaires anonymously, and the surveys were collected afterwards. No renumeration or gifts were given to participants to facilitate their participation in the survey. Assistant pharmacists and pharmacy students fell under the exclusion criteria of the study. All questionnaires were checked manually, and questionnaires with >90% completion were included in the analysis. All the completed questionnaires were entered into Epi-data version 3.1, and the data were exported to SPSS Statistics version 23.0 (IBM; Chicago, IL, USA) for data analysis. Descriptive statistics and univariate analysis were performed (Pearson’s Chi-squared tests or Fisher’s exact tests were used when comparing proportions, while Student’s t-tests were utilized to assess the association between numerical values). During statistical analyses, the respondents were classified into two age groups (24–35 years and older than 35 years; see Section 1 in the final instrument), and the self-perceived knowledge of the respondents on infectious diseases and ABs (see QK1-3 in Section 2 of the final instrument) was expressed as a score of 0–3 for statistical evaluation (one point awarded for every “True” answer). To make the results more intelligible in the text and scoring, “strongly agree” or “agree” were classified as “agree” and “strongly disagree” or “disagree” were classified as “disagree.”

To find a possible correlation between different attitudes, a correlational analysis was done. From the attitude questions, just the most important ones (excluding the opinions) and those whose answers were given on the same five-point Likert scale range were used for analysis. The chosen items are seen in Supplementary Table S2–S4. First, data were tested for normality by the Kolmogorov–Smirnov and Shapiro–Wilk tests. All parameters were skewed and not normally distributed, so nonparametric analyses were used. The strength of linear relationship between the four domains (appropriate theoretical, practical, preventive attitude and subjective knowledge level) was analyzed by the Spearman correlation coefficient. Two-tailed tests were utilized to assess significance. A difference with *p*  <  0.05 was considered statistically significance.

### 4.3. Ethical Approval

The survey was conducted in accordance with the Declaration of Helsinki and national and institutional ethical standards. Ethical approval for the study protocol was obtained from the Human Institutional and Regional Biomedical Research Ethics Committee, University of Szeged (registration number: 3688).

## 5. Conclusions

This is the first study in Hungary about the knowledge, attitude and practice of community pharmacists regarding antibiotic use and infectious diseases. Altogether, Hungarian pharmacists have appropriate knowledge regarding antibiotics and antimicrobial therapy, and they realize the public health impact of the growing antimicrobial resistance. Their theoretical, practical and preventive attitude is very positive. They consider the health promotion of infection prevention and control and patient education as very important aspects of the pharmaceutical work. The only malpractice identified was that a quarter of them were found to dispense antibiotics without a medical prescription, although many of them are aware that this must not be done. Another remarkable result was the detection of the deep influencing role of patients on dispensing practice.

Community pharmacists possess the opportunity (contact with prescribers and patients), commitment and capability (knowledge about medicines), all of which are key components for successful behavioral change of the patients towards rational antibiotic use. With further training, their motivation can be enhanced. An outstanding outcome of our survey was that half of the pharmacists disagreed with the possibility of expanding the role of pharmacists toward therapy selection or administering immunization. An improved theoretical attitude was interrelated to better practical and preventive attitudes. However, patient demand is not influenced by a high theoretical knowledge. Once the knowledge-levels and attitudes (which are modifiable factors) of community pharmacists regarding inappropriate AB use have been assessed, there will be a chance to design specific and focused interventions to encourage prudent antimicrobial use in an outpatient setting. On one hand, basic level-microbiology and pharmacotherapy-based educational interventions should be undertaken to reduce gaps of knowledge. On the other hand, educational interventions that target compliance with a professional code of ethics and attitude-change could also result in marked improvements in the dispensing habits of community pharmacists. Improving community pharmacists’ dispensing attitude and skills can be achieved by methods such as small group snapshot teaching, creative mnemonics like the “WWHAM” (Who is the patient, What are the symptoms, How long have the symptoms been present, Action taken, Medication being taken, and their experience) or “ASMETOD” (Age/appearance, Self or someone else, Medication, Extra medicines, Time persisting, History, Other symptoms, Danger symptoms) through the use of leaflets that facilitate self-care conversations with patients, mobile phone applications, and interactive antibiotic resistance maps.

## Figures and Tables

**Table 1:** Demographic characteristics of the participants.

Characteristics	% (n=)
**Gender**	
Female	69.8% (n = 134)
Male	30.2% (n = 58)
**Age**	
24–35 years	58.9% (n = 113)
36–50 years	27.1% (n = 52)
over 50 years	14.0% (n = 27)
**Board-Certified specializations**	
No specializations (Pharm.D. only)	65.6% (n = 126)
BCPS (Pharm.D. and specialization) Specializations represented:	34.4% (n = 66)
Pharmacy operation and management	31.4% (n = 60)
Pharmacology, pharmacotherapy	1.0% (n = 2)
Pharmaceutical care	1.0% (n = 2)
Phytotherapy	1.0% (n = 2)

Pharm.D.: Doctor of Pharmacy, BCPS: board-certified pharmaceutical specialist.

**Table 2:** Questions regarding self-perceived knowledge of CPs.

	True % (n=)	False % (n=)	Don’t Know/Uncertain % (n=)
***QK1*** *My knowledge regarding the pharmacological aspects of antibiotic therapy are appropriate.*	**90.1%** (n = 173)	**2.1%** (n = 4)	**7.8%** (n = 15)
***QK2*** *My knowledge regarding the pathomechanism and prevention of infectious diseases are appropriate.*	**70.8%** (n = 136)	**12.5%** (n = 24)	**16.7%** (n = 32)
***QK3*** *My knowledge regarding bacterial resistance are appropriate.*	**67.2%** (n = 129)	**12.0%** (n = 23)	**20.8%** (n = 40)

**Table 3:** Ratio of antibiotic (AB) prescriptions compared to total prescription drug traffic of given pharmacies, according to respondents’ assessment (QU1).

0%–5.0%	9.9%
5.1%–10.0%	24.5%
10.1%–15.0%	21.5%
15.1%–20.0%	19.3%
20.1%–25.0%	12.5%
more than 25.0%	12.5%

**Table 4:** Responses of CPs regarding attitudes and professional responsibility.

*Questions Regarding Attitudes and Professional Responsibility* (QA)	SD	D	U/DK	A	SA
	Disagree			Agree	
**Theoretical attitude**					
**QTA3** *I agree with the current funding policy of the National Institute of Health Insurance Fund Management regarding antibiotics (detailed in Decree No.32/2004 (IV.26.) Regulation by Ministry of Health, Social and Family Affairs about health insurance supported pharmaceuticals and the amount of subsidy).*	**2.1%** (n = 4)	**21.9%** (n = 42)	**28.6%** (n = 55)	**44.3%** (n = 85)	**3.1%** (n = 6)
**QTA4** *Antibiotics are medicines of special importance.*	**2.1%** (n = 4)	**3.6%** (n = 7)	**0.5%** (n = 1)	**45.3%** (n = 87)	**48.4%** (n = 93)
**QTA5** *I may be held responsible for the non-prescription dispensing of antibiotics, as this is a public health risk.*	**0.5%** (n = 1)	**12.5%** (n = 24)	**2.1%** (n = 4)	**43.8%** (n = 84)	**41.1%** (n = 79)
**QTA6** *Inappropriate antibiotic therapy does not cause significant surplus health costs on an annual basis.*	**41.1%** (n = 79)	**35.9%** (n = 69)	**4.2%** (n = 8)	**12.5%** (n = 24)	**6.3%** (n = 12)
**QTA7** *Education regarding antibiotics and antibiotic resistance should be more prominent during university training.*	**1.0%** (n = 2)	**5.2%** (n = 10)	**1.0%** (n = 2)	**35.9%** (n = 69)	**56.8%** (n = 109)
**QTA8** *The use of antibiotics in animal husbandry as growth promoters is just as important (or more important) in the development of bacterial resistance as their inappropriate prescription/consumption in health care.*	**0%** (n = 0)	**8.3%** (n = 16)	**12.5%** (n = 24)	**40.6%** (n = 78)	**38.5%** (n = 74)
**QTA9** *I consider it important to become acquainted with the antibiotics of the current drug pool and those newly licensed on the market.*	**0%** (n = 0)	**5.2%** (n = 10)	**1.0%** (n = 2)	**31.3%** (n = 60)	**62.5%** (n = 120)
**Practical attitude**					
**QPA2** *Patients are mostly receptive of my advice during dispensing, they welcome it.*	**3.1%** (n = 6)	**12.5%** (n = 24)	**5.2%** (n = 10)	**64.1%** (n = 123)	**15.1%** (n = 29)
**QPA3** *For patients requesting antibiotics without a prescription and are probably not in need of antibiotic therapy, I feel obligated to inform and educate them.*	**0%** (n = 0)	**12.0%** (n = 23)	**2.6%** (n = 5)	**53.1%** (n = 102)	**32.3%** (n = 62)
**QPA4** *There are several occasions when more time is needed to educate patients because doctors have not done this properly.*	**0%** (n = 0)	**6.3%** (n = 12)	**7.8%** (n = 15)	**52.6%** (n = 101)	**33.3%** (n = 64)
**QPA5** *The personality and behavior of patients significantly influences my dispensing practices.*	**17.2%** (n = 33)	**34.4%** (n = 66)	**4.7%** (n = 9)	**29.2%** (n = 56)	**14.6%** (n = 28)
**QPA6** *I offer probiotics for the patients purchasing a prescribed antibiotic.*	**0%** (n = 0)	**7.3%** (n = 14)	**0.5%** (n = 1)	**46.4%** (n = 89)	**45.8%** (n = 88)
**QPA7** *I detail the proper use of antibiotics when counselling the patient.*	**0%** (n = 0)	**2.0%** (n = 4)	**0%** (n = 0)	**36.5%** (n = 70)	**61.5%** (n = 118)
**Preventive attitude**					
**QPrA1** *The media devotes enough energy to disseminate information on infectious diseases.*	**37.0%** (n = 71)	**51.6%** (n = 99)	**2.6%** (n = 5)	**8.9%** (n = 17)	**0%** (n = 0)
**QPrA2** *Appropriate patient education would effectively reduce the incidence of infectious diseases.*	**0%** (n = 0)	**6.3%** (n = 12)	**1.6%** (n = 3)	**56.8%** (n = 109)	**35.4%** (n = 68)
**QPrA3** *As I am in direct contact with patients on a daily basis, I have the opportunity to influence their approach to infectious diseases.*	**0.5%** (n = 1)	**6.3%** (n = 12)	**1.6%** (n = 3)	**65.6%** (n = 126)	**26.0%** (n = 50)
**QPrA4** *During my work as a pharmacist, I not only have to make therapeutic decisions about acute infection, but I also have to provide lifestyle advice to the patient.*	**0%** (n = 0)	**2.6%** (n = 5)	**4.7%** (n = 9)	**53.1%** (n = 102)	**39.6%** (n = 76)
**QPrA5** *Proper use of antibiotics would be greater if pharmacists had time to perform their pharmacological care duties.*	**0.5%** (n = 1)	**15.6%** (n = 30)	**4.7%** (n = 9)	**53.1%** (n = 102)	**26.0%** (n = 50)
**Professional attitude**					
**QPh1** *Pharmacists should be authorized to perform the task of selecting the therapy in case of proven uncomplicated infections.*	**8.3%** (n = 16)	**33.9%** (n = 65)	**4.2%** (n = 8)	**39.1%** (n = 75)	**14.6%** (n = 28)
**QPh2** *After appropriate training, pharmacists could also perform the task of administering vaccines.*	**21.4%** (n = 41)	**32.8%** (n = 63)	**6.3%** (n = 12)	**30.2%** (n = 58)	**9.4%** (n = 18)
**QPh3** *Medicine-related counseling of community pharmacists is just as important as the physician’s recommendations.*	**2.6%** (n = 5)	**4.2%** (n = 8)	**0%** (n = 0)	**33.9%** (n = 65)	**59.4%** (n = 114)

Abbreviations: **A**: agree; **D**: disagree; **U/DK**: don’t know/unsure of the answer; **SA**: strongly agree; **SD**: strongly disagree; **QTA**: question related to theoretical attitude; **QPA**: question related to practical attitude; **QPrA:** question related to preventive attitude; and **QPh**: question related to pharmaceutical profession.

## Supplementary Materials

The following are available online at https://www.mdpi.com/2079-6382/9/2/41/s1. Supplementary material S1: questionnaire in English, Supplementary material S2: questionnaire in Hungarian, Supplementary material S3: including Supplementary Section S1 (Sample size calculation), Section S2 (Questionnaire development, reliability and scoring), Supplementary Table S1 (Item-total statistics related to internal consistency of the questionnaire instrument), S2 (Correlation of theoretical attitude with practical and preventive attitude according to Spearman correlational coefficient), S3 (Correlation of self-perceived knowledge with theoretical, practical and preventive attitude according to Spearman correlational coefficient) and S4 (Correlation of preventive attitude with practical attitude according to Spearman correlational coefficient).
