# Supplementary material for: Knowledge, Attitude and Practice of Community Pharmacists Regarding Antibiotic Use and Infectious Diseases: A Cross-Sectional Survey in Hungary (KAPPhA-HU)
Source: Antibiotics (Basel). 2020 Jan 21;9(2):41. doi: 10.3390/antibiotics9020041 (PMC7168197; doi:10.3390/antibiotics9020041)
Supplement: Supplementary file 1 [file antibiotics-09-00041-s001.zip › Supplementary material S2.pdf]

Supplementary material S2

Antibiotikum felhasználással kapcsolatos ismeretek és attitűdök

(Kérdőív gyógyszerészek részére)

A kérdőív kitöltése önkéntes és névtelen. Kérjük, válaszait a megfelelő szám bekarikázásával vagy szövegesen jelezze!

SZEMÉLYES ADATOK

1. Mi az Ön neme?
1. férfi
2. nő

2. Születési éve: ……………………………

3. Jelölje meg az Ön jelenlegi szakképzettségét!
1. gyógyszerész
2. szakgyógyszerész (is), éspedig: …………………………………………………..

4. Gyógyszertár helye (település): ……………………………………………………

ANTIBIOTIKUM FELHASZNÁLÁSRA VONATKOZÓ KÉRDÉSEK

5. Ön szerint probléma-e az antibiotikumok helytelen használata?
1. igen
2. nem

6. A gyógyszertárban megforduló betegek átlagosan hány százaléka szeretne antibiotikumra vonatkozó vényt kiváltani?
1. 5%
2. 10%
3. 15%
4. 20%
5. 25%
6. több, mint 25%

7. Milyen gyakran ad ki vény nélkül antibiotikumot az azt kérő páciensnek?
1. soha
2. az antibiotikum vásárlások kevesebb, mint 1%-ában
3. az antibiotikum vásárlások 1-5%-ában
4. az antibiotikum vásárlások 5-25%-ában
5. az antibiotikum vásárlások több, mint 25%-ában
6. egyéb esetben, éspedig: ………………………………………………………………………..

8. Az alábbi állításokról döntse el, hogy igazak-e (Önre)! Kérjük, hogy válaszait a megfelelő szám bekarikázásával jelölje!

	Igaz	Hamis	Nem tudom
A szakmai ismereteim az antibakteriális terápiára vonatkozóan megfelelőek.	1	2	7
A fertőző betegségek patomechanizmusával és prevenciójával kapcsolatos ismereteim megfelelőek.	1	2	7
A bakteriális rezisztenciával kapcsolatos ismereteim megfelelőek.	1	2	7
Problémának tartom, hogy van olyan gyógyszerész, aki vény nélkül is kiad vényköteles antibiotikumot a betegeknek.	1	2	7

Supplementary material S2

9. **Kérjük, jelölje meg, hogy milyen mértékben ért egyet az alábbi állításokkal! Válaszait a megfelelő szám bekarikázásával jelölje!**

	Egyáltalán nem ért egyet	Nem ért egyet	Egyet ért	Teljesen egyet ért	Nem tudja/ bizonytalan
Bizonyítottan szövődménymentes fertőzések esetén a gyógyszerészek is elláthatnák a terápia kiválasztásának feladatát.	1	2	3	4	7
A média elég energiát fordít a fertőző betegségekkel kapcsolatos ismeretterjesztésre.	1	2	3	4	7
A gyógyszerészek tanácsai a gyógyszerkiadás során legalább olyan fontosak, mint az orvos utasításai.	1	2	3	4	7
A gyógyszerészek is elláthatnák a védőoltások beadásának feladatát (megfelelő képzés után).					
Egyetértek a jelenleg hatályban lévő antibiotikumokra vonatkozó finanszírozási szabályokkal (lásd 1/2003. (I. 21.) ESzCsM rendelet a társadalombiztosítási támogatással rendelhető gyógyszerekről és a támogatás összegéről).	1	2	3	4	7
Az antibiotikumok kiemelt jelentőségű gyógyszerek.	1	2	3	4	7
Járványügyi kockázatot okozok és felelősség terhel, ha vény nélkül adok ki antibiotikumot.	1	2	3	4	7
A megfelelő betegoktatás hatékonyan csökkentené a fertőző betegségek előfordulását.	1	2	3	4	7
Mivel napi szinten közvetlen kapcsolatba kerülök a betegekkel, lehetőségem van arra, hogy befolyásoljam a fertőző betegségekkel kapcsolatos szemléletmódjukat.	1	2	3	4	7
Tanácsaimra a betegek többnyire fogékonyak, örömmel fogadják őket.	1	2	3	4	7
A nem megfelelően választott antibiotikum terápia éves szinten nem okoz jelentős többletköltséget az egészségügynek.	1	2	3	4	7
Az egyetemi képzés alatt lehetne hangsúlyosabb az antibiotikumok felhasználásához kapcsolódó oktatás.	1	2	3	4	7
Antibiotikumot vény nélkül kérő, antibiotikus terápiára feltehetőleg nem szoruló beteg esetén kötelességemnek érzem a felvilágosítást és a betegoktatást.	1	2	3	4	7
Sokszor azért kell több időt fordítani a betegek felvilágosítására, mert az orvosok ezt nem végezték el megfelelően.	1	2	3	4	7
Az antibiotikumok használata az állattenyésztésben, mint hozamfokozó szerek, legalább olyan fontos (vagy fontosabb) tényezője a bakteriális rezisztencia kialakulásának, mint a nem megfelelő indikációban történő felírásuk/fogyasztásuk az egészségügyben.	1	2	3	4	7

A betegek temperamentuma alapvetően befolyásolja a gyógyszerkiadási szokásaimat.	1	2	3	4	7
Az antibiotikumok megfelelő felhasználása nagyobb arányú lenne, ha a gyógyszerészeknek lenne ideje ellátni a gyógyszerészi gondozási feladataikat.	1	2	3	4	7
Gyógyszerészi munkám során nemcsak az akutan fennálló fertőzéssel kapcsolatban kell terápiás döntést hoznom, hanem életmódi tanácsokkal is el kell látnom a beteget.	1	2	3	4	7

10. Kérjük, hogy az alábbi kérdésekre adott válaszait a megfelelő szám bekarikázásával jelölje!

	**Mindig fontosnak tartom**	**Általában fontosnak tartom**	**Általában nem tartom fontosnak**	**Soha nem tartom fontosnak**	**Nem tudja/ bizonytalan**
Mennyire tartja fontosnak a probiotikumok ajánlását az antibiotikumot kiváltó betegnek és/vagy hozzátartozójának?	1	2	3	4	7
Mennyire tartja fontosnak, hogy kitérjen az antibiotikumok helyes felhasználásának minden részletére a beteg tájékoztatása során?	1	2	3	4	7
Mennyire tartja fontosnak, hogy megismerje a gyógyszerkincsben jelenleg található antibiotikumokat, illetve a piacon újonnan megjelenő termékeket?	1	2	3	4	7

KÖSZÖNJÜK, HOGY KITÖLTÖTTE A KÉRDŐÍVET!
