# Supplementary material for: Knowledge, Attitude and Practice of Community Pharmacists Regarding Antibiotic Use and Infectious Diseases: A Cross-Sectional Survey in Hungary (KAPPhA-HU)
Source: Antibiotics (Basel). 2020 Jan 21;9(2):41. doi: 10.3390/antibiotics9020041 (PMC7168197; doi:10.3390/antibiotics9020041)
Supplement: Supplementary file 1 [file antibiotics-09-00041-s001.zip › Supplementary Material S3.pdf]

Supplementary material S3

Supplementary Section S1. Sample size calculation

Hungary is in East-Central Europe with an area of 93,030 km^2 and an estimated population of 9,772,756 people (population density: 105.1/km^2, similar to the average in the European Union). Almost 100% of people have access to healthcare delivery and the 27.9% are pensioners. The number of graduated pharmacists (holders of the Pharm.D. degree) in the country is 12,799 (1.31/1000 inhabitants), while the number of pharmacists working in community pharmacies is 5575 (4566 full-time, 1027 part-time or as substitute pharmacists; 0.57/1000 inhabitants); the number of registered community pharmacies in the country is 2295, with most of the pharmacies are operating with one (21.5%) or two (36.6%) pharmacists (based on the data of the Hungarian National Healthcare Services Center, 2019) [1].

The study population comprised of CPs, as well as pharmacists attending post-gradual training courses aiming CPs throughout Hungary. Sample size for the population of 5575 CPs was calculated by using the Raosoft sample size calculator, based on the formula below (1): population was N=5575, x was confidence interval of 95%, E was the a margin of error set at 5% and the expected response rate set at 90%, based on our previous report aiming CPs in the Southern Great Plain of Hungary [2]. The minimum sample size of n=182 participants (n=136 with an added contingency of 25% for non-responders and inappropriate responses) was set for the completion of this survey.

$$\boldsymbol{n} = \boldsymbol{N}\frac{x}{(N-1)E2+x} \quad (1)$$

Supplementary Section S2. Questionnaire development, reliability and scoring

Based on the literature search, the questionnaire aiming CPs was adapted in accordance with local population characteristics and the questionnaires were developed in English and Hungarian languages. The questionnaire was then reviewed and assessed by five faculty members (with extensive experience in survey research), fifteen CPs and an infectious disease physician for content and face validation and to ensure the clarity and ease of use during the survey. Minor modifications (rewording, reformatting or reordering of questions) were performed based on the comments of the abovementioned panel. In addition, quantitative content validity of the instrument was ascertained, based on Lawshe's Content Validity, where a content validity ratio (CVR) was calculated for each item; items with a CVR score of ≥0.7 were selected acceptable (all items reported with CVR scores above 0.7) [3]. Pharmacists included in the pilot study were excluded from the main study.

The internal consistency of the data was assessed by calculating Cronbach's α value regarding the third segment (corresponding to the construct of CPs' personal responsibility regarding AB resistance) of the questionnaire; during the analysis, **QPrA1** was removed, as it did not correspond with the measured construct, while the scales of **QPA5** and **QTA6** were reversed, due to the wording of the question. The reliability of the questionnaire was evaluated by the Cronbach's α value, which was 0.72, indicating acceptable internal consistency [4], detailed item-total statistics are presented in the Supplementary Table S1 ; the corrected item-total correlation represent the correlation between the item and the composite score of all other items (ideally ranging between 0.3-0.7). In addition, fifteen (n=15) pharmacists were asked to complete the questionnaire twice, after a period of 1-2 months to perform test-retest analysis; the intraclass correlation coefficient for the re-take of questionnaires was also very good (0.90, 95% CI: 0.88-0.93).

Supplementary material S3

Supplementary Table 1. Item-total statistics related to internal consistency of the questionnaire instrument

Questions regarding attitudes and professional responsibility (**QA**)	*Corrected item-total correlation*	*Cronbach's α value if item deleted*
**QTA3**	0.429	0.653
**QTA4**	0.393	0.687
**QTA5**	0.381	0.672
**QTA6 (reversed)**	0.365	0.674
**QTA7**	0.319	0.670
**QTA8**	0.336	0.664
**QTA9**	0.332	0.692
**QPA2**	0.370	0.683
**QPA3**	0.332	0.676
**QPA4**	0.393	0.658
**QPA5 (reversed)**	0.317	0.667
**QPA6**	0.323	0.684
**QPA7**	0.282	0.698
**QPrA2**	0.303	0.679
**QPrA3**	0.278	0.692
**QPrA4**	0.486	0.643
**QPrA5**	0.369	0.672
**QPh1**	0.433	0.653
**QPh2**	0.398	0.665
**QPh3**	0.398	0.665

Supplementary material S3

Supplementary Table S2. Correlation of theoretical attitude with practical and preventional attitude according to Spearman correlational coefficient (*correlation is significant at the p<0.05 level; **correlation is significant at the p<0.01 level)

		THEORETICAL ATTITUDE					
		Non-prescription dispensing of antibiotics by others is a problem	*Consideration of antibiotics as medicines of special importance*	*If I dispense a non-prescription antibiotic, I am responsible for the public health risk*	*Inappropriate antibiotic therapy causes surplus cost in health care*	*Antibiotic misuse in animal husbandry has a role in antibiotic resistance*	*Important to get to know currently available and newly licensed antibiotics*
PRACTICAL ATTITUDE	*Dispensing non-prescription antibiotics less often*	0,242**	0,030	0,191**	0,103	-0,011	0,269**
	Patients accept my advice	0,010	-0,118	0,020	0,171*	0,167*	0,179*
	Informing and educating patients requesting antibiotics without prescription	0,232**	0,177*	0,229**	0,255**	0,227**	0,185*
	Patients' temperament does not influence dispensing practices	-0,010	-0,043	-0,048	-0,019	-0,032	0,101
	Recommend probiotics for antibiotics	-0,018	0,047	-0,065	0,005	0,022	0,047
	Detailed counselling about the proper use of antibiotics	0,071	-0,025	0,027	-0,095	-0,134	0,281**
PREVENTIONAL ATTITUDE	*Media does not devote enough energy to disseminate information on infectious diseases*	-0,055	-0,134	0,050	0,068	0,033	-0,067
	Proper patient education can effectively reduce the incidence of infectious diseases	0,138	0,126	0,347**	0,266**	0,105	0,122
	Pharmacists can influence the patients' approach about infectious diseases	0,161*	0,105	0,285**	0,098	0,188**	0,124
	Advices on healthy lifestyle must be given to patients, as well	0,227**	0,129	0,175*	0,049	0,179*	0,165*

Supplementary material S3

Supplementary Table S3. Correlation of self-perceived knowledge with theoretical, practical and preventional attitude according to Spearman correlational coefficient (*correlation is significant at the p<0.05 level; **correlation is significant at the p<0.01 level)

		SELF-PERCEIVED KNOWLEDGE		
		Antibiotic therapy	*Patomechanism and prevention of infectious diseases*	*Bacterial resistance*
THEORETICAL ATTITUDE	*Non-prescription dispensing of antibiotics by others is a problem*	-0.068	-0.035	0.219**
	Consideration of antibiotics as medicines of special importance	0.125	-0.006	0.008
	If I dispense a non-prescription antibiotic, I am responsible for the public health risk	-0.013	0.008	-0.047
	Inappropriate antibiotic therapy causes surplus cost in health care	-0.140	-0.042	-0.107
	Antibiotic misuse in animal husbandry has a role in antibiotic resistance	-0.069	-0.003	-0.083
	Important to get to know currently available and newly licensed antibiotics	0.078	0.187**	0.111
PRACTICAL ATTITUDE	*Dispensing non-prescription antibiotics less often*	0.005	0.223**	0.222**
	Patients accept my advice	-0.061	-0.008	0.011
	Informing and educating patients requesting antibiotics without prescription	-0.009	0.077	0.080
	Patients' personality and behavior does not influence dispensing practices	0.093	0.118	0.128
	Recommend probiotics for antibiotics	0.063	0.015	-0.023
	Detailed counselling about the proper use of antibiotics	0.057	0.099	-0.054
PREVENTIONAL ATTITUDE	*Media does not devote enough energy to disseminate information on infectious diseases*	-0.033	-0.254**	-0.092
	Proper patient education can effectively reduce the incidence of infectious diseases	0.050	-0.124	-0.059
	Pharmacists can influence the patients' approach about infectious diseases	0.001	-0.194**	-0.054
	Advices on healthy lifestyle must be given to patients, as well	-0.019	-0.108	0.174*

Supplementary material S3

Supplementary Table S4. Correlation of preventional attitude with practical attitude according to Spearman correlational coefficient (*correlation is significant at the p<0.05 level; **correlation is significant at the p<0.01 level)

		PREVENTIONAL ATTITUDE			
		Media does not devote enough energy to disseminate information on infectious diseases	*Proper patient education can effectively reduce the incidence of infectious diseases*	*Pharmacists can influence the patients' approach about infectious diseases*	*Advices on healthy lifestyle must be given to patients, as well*
**PRACTICAL ATTITUDE**	*Dispensing non-prescription antibiotics less often*	-0,166*	-0,019	0,016	0,039
	Patients accept my advice	-0,054	0,028	0,244**	0,096
	Informing and educating patients requesting antibiotics without prescription	-0,029	0,226**	0,308**	0,418**
	Patients' personality and behavior does not influence dispensing practices	-0,098	0,051	0,178*	0,084
	Recommend probiotics for antibiotics	-0,174*	0,139	0,067	0,121
	Detailed counselling about the proper use of antibiotics	-0,180*	0,060	0,044	0,011

Supplementary material S3

References

1. Nagy, V.; Sohajda, A.; Hankó, Z. How is the current staffing situation in community pharmacies? (article in Hungarian). *Gyógyszerészet 63*, 459–466.

2. Gajdács, M.; Paulik, E.; Szabó, A. The opinions of community pharmacists towards antibiotic use and resistance (article in Hungarian). *Acta Pharmaceutica Hungarica 88*, 249–252.

3. Polit, D.F.; Beck, C.T.; Owen, S.V. Is the CVI an acceptable indicator of content validity? Appraisal and recommendations. *Res Nurs Health* **2007**, *30*, 459–467.

4. Gliem, J.A.; Gliem, R.R. Calculating, Interpreting, And Reporting Cronbach's Alpha Reliability Coefficient For Likert-Type Scales. **2003**.
