# Supplementary material for: Knowledge, Attitude and Practice of Community Pharmacists Regarding Antibiotic Use and Infectious Diseases: A Cross-Sectional Survey in Hungary (KAPPhA-HU)
Source: Antibiotics (Basel). 2020 Jan 21;9(2):41. doi: 10.3390/antibiotics9020041 (PMC7168197; doi:10.3390/antibiotics9020041)
Supplement: Supplementary file 1 [file antibiotics-09-00041-s001.zip › Supplementary material S1.pdf]

Supplementary material S1

KNOWLEDGE AND ATTITUDE RELATED TO ANTIBIOTIC UTILIZATION

(Questionnaire for pharmacists)

Filling out the questionnaire is completely VOLUNTARY and ANONYMOUS.
Please, circle the answers or write a text where needed!

PERSONAL INFORMATION

1. What is your gender?
1. male
2. female

2. Year of birth: ……………………………

3. What is your specialization?
1. pharmacist without any specialization
2. pharmacist specialized in: ………………………………………………….

4. **Place of the pharmacy (name of the settlement)**: ……………………………………………………

QUESTIONS FOR ANTIBIOTIC UTILIZATION

5. Do you think that improper use of antibiotics is a problem?
1. yes
2. no

6. How much percentage of patients would like to purchase a prescribed antibiotic in your pharmacy compared to total prescription drug traffic?
1. 5%
2. 10%
3. 15%
4. 20%
5. 25%
6. more than 25%

7. How often do you dispense antibiotic when a patient requires it without medical prescription?
1. never
2. less, than 1% of all antibiotic purchases
3. in 1-5% of all antibiotic purchases
4. in 5-25% of all antibiotic purchases
5. more than 25% of all antibiotic purchases
6. other: ……………………………………………………………………….

8. Decide whether the following statements are true or not (about you)! Please circle the correct answer!

	**True**	**False**	**Don’t know**
My knowledge regarding the pharmacological aspects of antibiotic therapy are appropriate.	1	2	7
My knowledge regarding the patomechanism and prevention of infectious diseases are appropriate.	1	2	7
My knowledge regarding bacterial resistance is appropriate.	1	2	7
I believe that it is problematic that there are pharmacists who dispense antibiotic when the patient require it without prescription.	1	2	7

Supplementary material S1

9. **To what extent do you agree or disagree with the following statements! Please, circle the appropriate number!**

	Totally disagree →				Totally agree
Pharmacists should be authorized to perform the task of selecting the therapy in case of proven uncomplicated infections.	1	2	3	4	5
The media devotes enough energy to disseminate information on infectious diseases.	1	2	3	4	5
Medicine-related counselling of community pharmacists is just as important as the physician's recommendations.	1	2	3	4	5
Pharmacists may also perform the task of administering vaccines (after proper training).	1	2	3	4	5
I agree with the current funding policy of the National Institute of Health Insurance Fund Management regarding antibiotics (detailed in Decree No.32/2004 [IV.26.] Regulation by Ministry of Health, Social and Family Affairs about health insurance supported pharmaceuticals and the amount of subsidy).	1	2	3	4	5
Antibiotics are medicines of special importance.	1	2	3	4	5
I may be held responsible for the non-prescription dispensing of antibiotics, as this is a public health risk.	1	2	3	4	5
Appropriate patient education would effectively reduce the incidence of infectious diseases.	1	2	3	4	5
As I am in direct contact with patients on a daily basis, I have the opportunity to influence their approach to infectious diseases.	1	2	3	4	5
Patients are mostly receptive of my advice during dispensing, they welcome it.	1	2	3	4	5
Inappropriate antibiotic therapy does not cause significant surplus health costs on an annual basis.	1	2	3	4	5
Education regarding antibiotics and antibiotic resistance should be more prominent during university training.	1	2	3	4	5
For patients requesting antibiotics without prescription and are probably not in need of antibiotic therapy, I feel obligated to inform and educate them.	1	2	3	4	5
There are several occasions when many times more time is needed to educate patients because doctors have not done this properly.	1	2	3	4	5

Supplementary material S1

The use of antibiotics in animal husbandry as growth promoters is just as important (or more important) in the development of bacterial resistance as their inappropriate prescription/consumption in health care.	1	2	3	4	5

	**Totally disagree**				**Totally agree**
The personality and behavior of patients significantly influences my dispensing practices.	1	2	3	4	5
Proper use of antibiotics would be greater if pharmacists had time to perform their pharmacological care duties.	1	2	3	4	5
During my work as a pharmacist, I not only have to make therapeutic decisions about acute infection, but I also have to provide lifestyle advice to the patient.	1	2	3	4	5
I offer probiotics for the patients purchasing a prescribed antibiotic.	1	2	3	4	5
I detail the proper use of antibiotics when counselling the patient.	1	2	3	4	5
I consider it important to become acquainted with the antibiotics of the current drug pool and those newly licensed on the market.	1	2	3	4	5

THANK YOU FOR ANSWERING THE QUESTIONS!
